# Lentiviral delivery of novel fusion protein IL12/FasTI for cancer immune/gene therapy

**DOI:** 10.1371/journal.pone.0201100

**Published:** 2018-07-25

**Authors:** Xi Yang, Xianzhong Yu, Yanzhang Wei

**Affiliations:** Department of Biological Sciences, Clemson University, Clemson, SC, United States of America; George Mason University, UNITED STATES

## Abstract

Many of the cytokine-based cancer immunotherapies are hindered by the devastating side effects of systemic delivery of the cytokines. To address this problem, we previously described a novel approach to locally achieve high doses of interleukin-12 (IL-12) in tumors and demonstrated that bi-functional fusion protein mIL-12/FasTI expressed by stable clones of TC-1 cells efficiently suppressed tumor proliferation by activating natural killer (NK) cells and other cytolytic killer cells and sending apoptotic signals into tumor cells. In the present study, we employed a lentiviral vector-based gene delivery system to deliver this fusion construct directly into tumor cells. We show that lentiviral vector efficiently delivers the fusion constructs into Hela cells *in vitro* as assayed by RT-PCR and immunohistochemistry (IHC). We also confirm that fusion protein mIL-12/FasTI delivered by the viral vector significantly enhanced killer cell activation, increased caspase-3 activity and decreased tumor growth *in vitro*. This study offers a further step for fusion protein cancer therapy for cancer patients.

## Introduction

Natural killer (NK) cells are specialized lymphocytes capable of targeting virus-infected cells and malignant cells such as tumor cells. Unlike cytotoxic T cells (CTL), NK cells recognize potential target cells without the need for immunization or pre-activation [1]. The regulation of NK cell activation requires a variety of cell surface activating and inhibitory receptors. Importantly, NK cells are inhibited by receptors that recognize MHC class I molecules; therefore, normal cells expressing high levels of MHC class I can be protected from NK cell-mediated cytotoxicity [2]. NK cell activation also requires engagement of pro-inflammatory cytokines, particularly interleukin-12 (IL-12), IL-15 and type I interferons (IFN), which enhance NK cell proliferation as well as promote NK cell cytotoxicity and/or production of IFN-γ [3, 4]. Recent evidence suggests that NK cells, besides their direct cytolytic effect against tumor cells, may also shape the adaptive immune response toward a T helper1 (Th1) profile, which is thought to favor antitumor responses [5]. Despite the powerful cytotoxic activity of NK cells, the tumor microenvironment of advanced tumors can interfere with NK cell activation pathways or the complex receptor array that regulate NK cell activation and anti-tumor effects. One major challenge for effective cancer therapy is how to reshape the tumor microenvironment to favor NK cells and other killer cells.

IL-12 is a heterodimeric protein composed of covalently linked p35 and p40, which is majorly derived from activated antigen presenting cells (APC). It was first recognized as “natural killer-stimulating factor” by Trinchieri and colleagues in 1989. By enhancing the production of IFN-γ, proliferation and cytotoxic ability of NK cells and T cells, IL-12 bridges innate and adaptive immunity. In addition, IL-12 induces an antiangiogenic program mediated by IFN-γ and lymphocyte-endothelial cell cross-talk [6, 7]. The antitumor and anti-metastatic activities of IL-12 have been extensively demonstrated in many murine models and clinical studies. However, systemic administration of IL-12 in patients is limited by severe toxicity. Additionally, the immune suppression-dominated microenvironment in advanced tumors is a major obstacle limiting the efficacy of IL-12-based therapies.

Fas (CD95/APO-1) is a cell surface death receptor of tumor necrosis factor (TNF) receptor superfamily. Binding of Fas ligand (FasL) induces Fas trimerization, which results in recruitment of adapter protein Fas-associated death domain (FADD) through DD-DD interactions. The death-effector domain of FADD then recruits pro-caspase-8/10 to the receptor, resulting in the formation of death-inducing signaling complex (DISC) [8, 9]. DISC activates effector caspases such as caspase-3 that in turn cleave a restricted set of targets proteins and are responsible for cell apoptosis.

In our previous study, we have demonstrated that mouse IL-12/FasTI, a bi-functional fusion protein containing mouse IL-12 and Fas transmembrane/intracellular domains (FasTI), can simultaneously enhance NK cell activity and induce apoptosis of tumor cells in a tumor microenvironment, resulting in effective tumor cell elimination *in vitro* [10]. To further confirm the antitumor efficacy of fusion protein IL-12/FasTI in a therapeutic setting, a high-efficient gene delivery method is in demand. Lentiviruses are a subgroup of the retrovirus family, which have been developed mostly from simian immunodeficiency virus (SIV) and human immunodeficiency virus type I (HIV-1) [11]. The design of lentivirus-based expression vectors allows high-level expression of recombinant proteins in dividing and non-dividing mammalian cells. In the present study, lentiviral vectors, containing the cDNA sequence of fusion gene mIL12/FasTI (pLenti7.3/ mIL12/FasTI) and the control gene (pLenti7.3/IL12, pLenti7.3/Fas), were established through advanced molecular cloning. The pLenti7.3/V5-TOPO lentiviral expression vector contains two new elements to yield cell-specific and high performance delivery: the WPRE (Woodchuck Posttranscriptional Regulatory Element) and cPPT (Polypurine Tract), which can produce at least a four-fold increase in viral titer [12, 13]. The titer of each viral clone was determined by transducing 293 cells. The transient gene expression of IL-12/FasTI, IL-12 and Fas via lenti-viral transduction was confirmed by both reverse-transcription PCR (RT-PCR) and immunohistochemistry (IHC). It was also demonstrated that the expression of IL-12/FasTI enhanced apoptosis levels, NK cell activity as well as overall cytotoxicity against tumor cells, comparing to IL-12 and Fas controls. Combined with high-efficient lentiviral expression system, our fusion protein strategies might serve as one potential option for cancer immuno/gene therapy in the future.

## Materials and methods

### Cells

293 cell line (ATCC No. CRL-1573) was cultured in Eagle's Minimum Essential Medium containing 10% fetal bovine serum and 100μg/ml gentamicin at 37°C with 5% CO_2_. 293FT (Thermo Fisher Scientific Cat# R700-07) were cultured in D-MEM medium containing 10% FBS supplemented with 0.1 mM MEM Non-Essential Amino Acids, 1 mM sodium pyruvate, 2 mM L-glutamine and 500μg/ml Geneticin as “selective antibiotics”) at 37°C with 5% CO_2_. Human cervical carcinoma Hela cells (ATCC No. CCL-2) were cultured in high-glucose DMEM containing 10% fetal bovine serum and 100μg/ml gentamicin at 37°C with 5% CO_2_. Human NK92 cells (ATCC No. CRL-2407) were cultured in RPMI1640 media containing 20% fetal bovine serum, 100 μg/ml gentamicin and 100 IU Interleukin-2 (IL-2) (NK media) at 37°C with 5% CO_2_.

### Construction of lentiviral vectors

Mouse *IL-12/FasTI* cDNA sequence was cloned from pcDNA3.1/IL-12/FasTI/Zeo(+) vector from previous study using ACCATGGGTCAATCACGCTAC as 5’ primer and CTCCAGACATTGTCCTTCATTTTC as 3’ primer. The optimal sequences for translation initiation (Kozak sequences) were included in the sequence. To enable TA cloning with pLenti7.3/V5-TOPO vector, *Taq* DNA polymerase was used for PCR to generate extruding “A” at both ends of PCR product. Four microliter purified PCR product of *IL-12/FasTI*, 1μl Salt solution and 1μl pLenti-TOPO vector (Invitrogen, USA) were then mixed and incubated at room temperature for 5 min for TOPO cloning reaction. Six ampicillin-resistant colonies from transformation were picked, and plasmid DNA for each colony was isolated using a QIAprep Spin miniprep kit (Qiagen). The plasmid was analyzed by restriction enzyme digest and DNA Sanger sequencing to confirm the presence and orientation of insert as well as the integrity of the vector.

Mouse *IL-12* cDNA sequence was cloned from pcDNA3.1/IL-12/Zeo(+) from previous study using ACCATGGGTCAATCACGCTAC as 5’ primer and CGTGGCTTCTTCTGCCAAAGCATG as 3’ primer. Mouse *Fas* cDNA sequence was cloned from pCMV-mFAS-His purchased from Sino Biological Inc., using ACCATGGTGTGGATCTGG as 5’ primer and CTCCAGACATTGTCCTTCATTTTC as 3’ primer. PLenti7.3/IL-12 and pLenti7.3/Fas were constructed by the same methodology following manufacturer’s instructions. pLenti7.3/IL-12 and pLenti7.3/Fas sequences were analyzed by DNA Sanger sequencing to confirm the presence and orientation of insert as well as the integrity of the vector.

### Production of lentiviral particles in 293FT cells

293FT cells were co-transfected with pLenti7.3/mIL-12/FasTI, pLenti7.3/mIL-12, or pLenti7.3/mFas, respectively, as well as lentiviral packing mix (containing three packing plasmids, pLP1, pLP2 and pLP/VSVG) using Lipofectamine 2000 (Invitrogen) as directed by the manufacturer’s instructions. Virus-containing supernatant of each pLenti expression construct was harvested 48–72 hours post-transfection directed by manufacturer’s instruction. Meanwhile, pLenti7.3/V5-GW/LacZ was used as a control in co-transfection to optimize expression conditions. Before proceeding to transduction and expression experiments, lentiviral stock of each construct was concentrated by using Lenti-X concentrator (ClonTech) and the viral titer was determined for further analyses. Lentiviral titration was determined by fluorescence-based cytometry of GFP positive cells following manufacturer’s instruction.

### RT-PCR

Three hundred thousand Hela cells were plated on 6-well-plate and incubated for 24 hour with Hela media. After the incubation, the media was removed and replaced with DMEM media containing 8μg/ml polybrene. Lentiviral particles of pLenti/IL-12/FasTI, pLenti/IL-12 and pLenti/Fas (Lent-IF, Lent-IL12, Lent-Fas) were added in plating media by1:10 dilution, and incubated for another 48 hour for transient transduction. Total RNA was extracted from each clone using an RNeasy Plus Mini kit following the manufacturer’s directions. RT-PCR was run with 2μg of the total RNA using Phusion RT-PCR kit following the manufacturer’s instruction (Thermo Fisher). To amplify a 1059-bp segment of IL-12/FasTI containing 522-bp of the mIL-12, the linker (GGTGGTGGTTCTGGTGGTGGTTCTGGTGGTGGTTCT), and the entire 495-bp of FasTI, a 5’ primer GCAGTGACATGTGGAATGGC and a 3’ primer CGGAATTCTCACTC CAGACATTGTCCTTCATTTTC were used. To amplify 180-bp portion of IL-12, a 5’ primer GGAAGCACGGCAGCAGAATA and a 3’ primer AACTTGAGGGAGAAGTAGGAATGG were used. To amplify 984-bp of Fas sequence, a 5’ primer ACCATGGTGTGGATCTGG and a 3’ primer CTCCAGACATTGTCCTTCATTTTC were used. All RT-PCR reactions included the β-actin housekeeping gene as loading control using a 5’primer ATGGGTCAGAAGGATTCCTATGTG and a 3’ primer CTTCATGAGGTAGTCAGTCAGGTC amplifying a 488-bp fragment.

### Immunohistochemistry (IHC)

Viral transduction was performed on Hela cells as described in RT-PCR. After transduction, cells of each group (Lent-IF, Lent-IL12, Lent-Fas) were cytospun onto glass slides by centrifuging at 2,000 rpm for 10 min at room temperature. After centrifugation the cells on the slides were fixed with 4% para-formaldehyde (PFA) solution for 10 min. The cells were then stained at room temperature for one hour with isotype rat IgG_2a_ antibody (R&D Systems, clone #54447) and/or anti-mouse IL-12/p35 antibody (R&D Systems, clone #45806), after a 1:100 dilution with sterile PBS. After washing, cells were treated with secondary antibody conjugated with HSS-HRP for 30 min at room temperature. DAB substrate-chromogen mixture was applied to the cells and incubated for 10–20 min. Slides were washed three times using fresh PBS, and then examined under a microscope (Olympus 1x70 fluorescent microscope).

### Coculture of human NK cells with lentiviral transduced cells

Human NK92 cells were plated with Hela cells, or lentiviral transduced Hela cells expressing IL-12/FasTI, IL-12 or Fas on 96-well plate at a ratio of 5:1 in 200 μl NK media for 48 hours. After co-cultural incubation, 100 μl of supernatant was removed for analysis of IFN-γ production using human IFN-γ ELISA reagent kit (Thermo Fisher Scientific) following manufacturer’s direction. The remaining media was removed, and suspension NK92 cells were carefully removed. The number of remaining tumor cells were determined using CellTiter 96 AQueous non-radioactive cell proliferation assay (Promega) following manufacturer’s directions. The results were analyzed using a one way ANOVA with Tukey’s post-test.

### Tumor cell apoptosis

One million cells of Hela cells or lentiviral transduced Hela cells expressing IL-12/FasTI, IL12 or Fas were first plated on a 6-well-plate and incubated for 24 hour. At this point one million NK92 cells were added to all the cells and incubated overnight for coculture. After removing suspension NK92 cells, apoptosis of the tumor cells was determined by a caspase 3 assay kit (abcam) following the manufacturer’s instruction.

### Statistical analysis

GraphPad software package was used to plot graphs and run statistical analysis. The statistical significance was represented as *p<0.05, **p<0.01, ***p<0.001.

## Results

### Lentiviral vector construction

**Fig 1:**
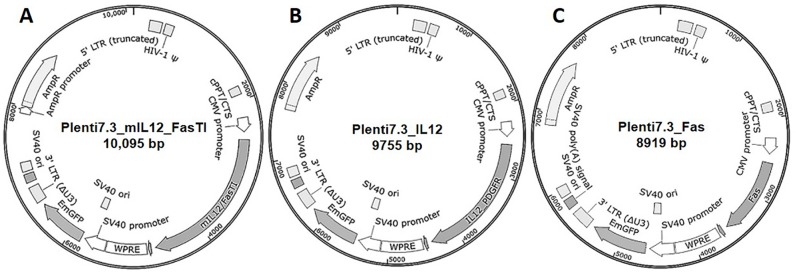
Constructs of lentivirus-based gene expression vectors. (a) Construct of Plenti7.3/mIL12_FasTI/V5-TOPO; (b) Construct of Plenti7.3/mIL12/V5-TOPO; (c) Construct of Plenti7.3/mFas/V5-TOPO.

Mouse *IL-12/FasTI* cDNA sequence was amplified from pcDNA3.1/IL-12/FasTI/Zeo(+) by PCR and inserted into lentiviral expression vector PLenti7.3/V5_TOPO using TA cloning. Control mouse *IL-12* and *Fas* sequence were also cloned into pLenti7.3/V5_TOPO vector. The resulting Lentiviral constructs, Lent-IF, Lent-IL12, and Lent-Fas, were analyzed by restriction enzyme digest and DNA Sanger sequencing for orientation of ligation and integrity of entire plasmid (Fig 1).

### Lentiviral titer determination

**Fig 2:**
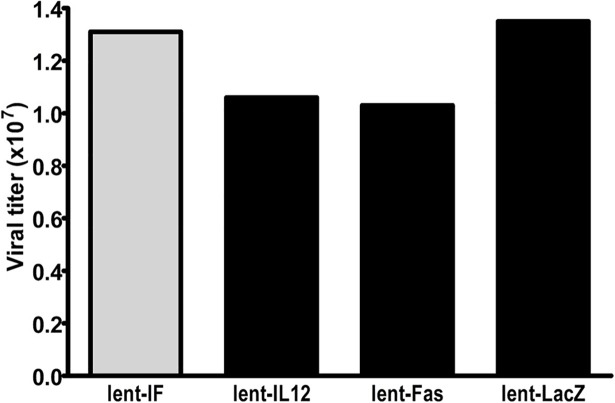
Lentiviral titer determination. Each virally transduced 293-Lent clones (Lent-IF/Lent-IL12/Lent-Fas/Lent-LacZ) were collected for EmGFP-based cytometry analysis. Viral titer of each lent-clone was calculated based on cytometry reading and represented.

293FT cells were co-transfected with Lent-IF, Lent-IL12, and Lent-Fas, respectively, and lentiviral packing mix using Lipofectamine 2000. Virus-containing supernatant of each Lent-construct was harvested 48–72 hours post-transfection. The lentiviral particles were first concentrated using Lenti-X^TM^ Concentrator (Clontech). Concentrated viral particles were then assayed to determine their viral titers. Emerald Green fluorescent protein (EmGFP) gene carried with the lentiviral vectors allows convenient determination of the lentiviral titers. 293 cells were transduced with Lent-IF, Lent-IL12, Lent-Fas and Lent-LacZ, respectively, for 48 hours, and virally transduced cells as well as un-transduced 293 cells were collected to determine percentage of EmGFP positive cells by Tali^TM^ image-based cytometry (Invitrogen). The percentages of fluorescent cells in Lent-IF, Lent-IL12, Lent-Fas and Lent-LacZ transductions are 58.1%, 47.2%, 45.9 and 60.2%. The titer was then calculated following the equation: titer = {(F × Cn) /V} × DF (F: The frequency of GFP-positive cells determined by cytometry; Cn: The total number of target cells infected; V: The volume of the inoculum; DF: The virus dilution factor). The titers are between 1.03 to 1.35x10^7^ /ml (Fig 2).

### Gene expression via lentiviral delivery system

**Fig 3:**
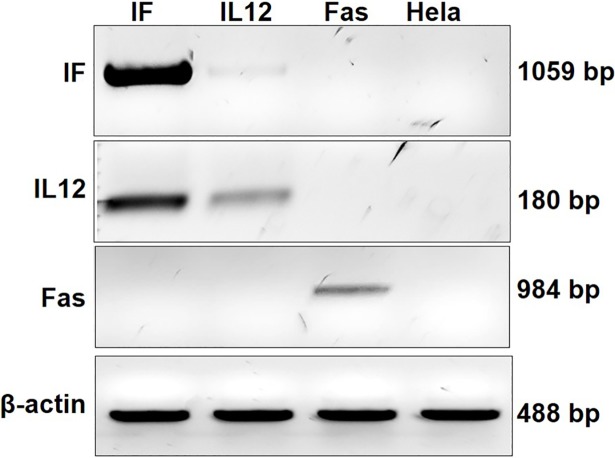
RT-PCR Confirmation of gene expression in Lent-clones. Total RNAs were isolated from Lent-clones and un-transduced Hela cells and a two-step RT-PCR was performed using Phusion RT-PCR kit (Thermo Scientific). Corresponding pairs of primers were used to amply different fragments. House-keeping gene β-actin expression was also included as controls.

**Fig 4:**
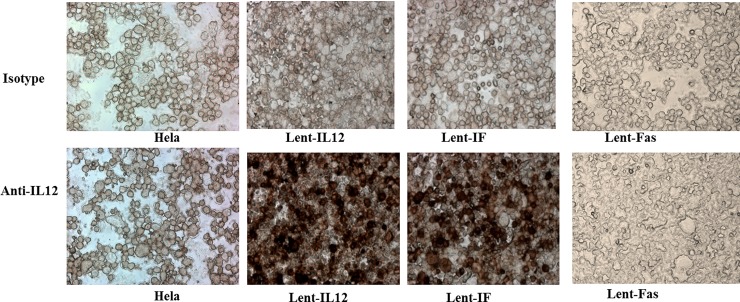
IHC staining of Lent-clones. Cells from Lent-clones and Hela cells were harvested and cytospun onto glass slides. After fixation, the cells were stained with anti-mouse IL-12 antibody or isotype control antibody followed by anti-mouse IgG secondary antibody conjugated with HRP. The cells were then treated with substrate DAB and observed under microscope.

Human cervical cancer Hela cells were transduced with Lent-IF, Lent-IL12, or Lent-Fas and RT-PCR and IHC were performed to determine the gene expressions. RT-PCR shows that after lentiviral transduction, all the three constructs, Lent-IF, Lent-IL12, and Lent-Fas are transcribed correctively (Fig 3; supporting information). IHC results demonstrated that IL12/Fas and IL-12 proteins are expressed on the cell surface (Fig 4). Fluorescent microscopic analysis showed that control Fas protein was also expressed on the cell surface (data not shown).

### Human NK cell activation

**Fig 5:**
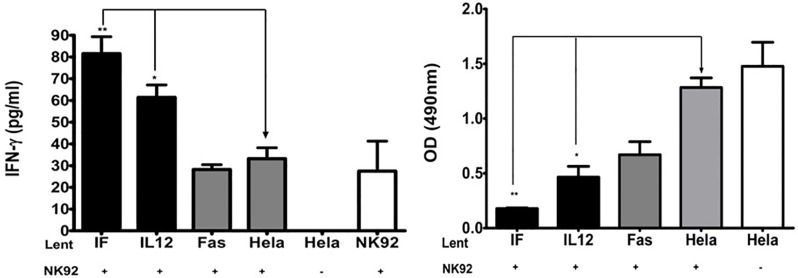
Activation of human NK92 cells. (a) IFN-γ ELISA. Cells of Lent-clones and Hela cells in triplicates were co-cultured with human NK92 cells and the supernatants were collected. IFN-γ activity in the supernatants was detected using Human IFN-γ ELISA Kit. The statistical analyses were conducted between the control (Hela+NK) and each Lent-clone (as the bars represented in the figure) using one-way analysis of variance (ANOVA) with Tukey’s post test. (*p<0.05; **p<0.01) (b) Cytotoxicity of NK92 cells. Cells of each lent-clone and Hela in triplicates were co-cultured with human NK92 cells. After co-culture, NK92 cells were removed and remaining tumor cells’ proliferation was measured with Promega’s CellTiter 96ueous nonRadioactive cell proliferation assay kit. The statistical analyses were conducted between the control (Hela+NK) and each virally transduced lent-clone using one-way analysis of variance (ANOVA) with the Tukey’s post test (*p<0.05; **p<0.01).

In order to determine if the bi-functional fusion protein IL-12/FasTI delivered by the lentiviral system can activate killer cells, human NK92 cells were cocultured with Hela cells transduced with Lent-IF, Lent-IL12, or Lent-Fas for 48 hrs. One hundred microliter supernatant was collected from each well and screened for IFN-γ production by ELISA. NK92 cells cocultured with Hela cells transduced with Lent-IF and Lent-IL12 showed significantly increased production of IFN-γ comparing to the controls (Fig 5A). While the IFN-γ production by NK92 cells cocultured with Hela cells transduced with Lent-IF is not statistically higher than that of NK92 cells cocultured with Hela cells transduced with Lent-IL12, the enhanced trend is clear. Interestingly, NK92 cells cocultured with Hela cells transduced with Lent-Fas produced significantly higher IFN-γ than control Hela cells. The cytotoxicity of tumor cells after co-culturing with NK92 cells were measured with MTS assay. NK92 cells co-cultured with Hela cells transduced with Lent-IF and Lent-IL12 killed much more tumor cells than the controls (Fig 5B). More importantly, Lent-IF is significantly more effective than Lent-IL12.

### Apoptosis induction

**Fig 6:**
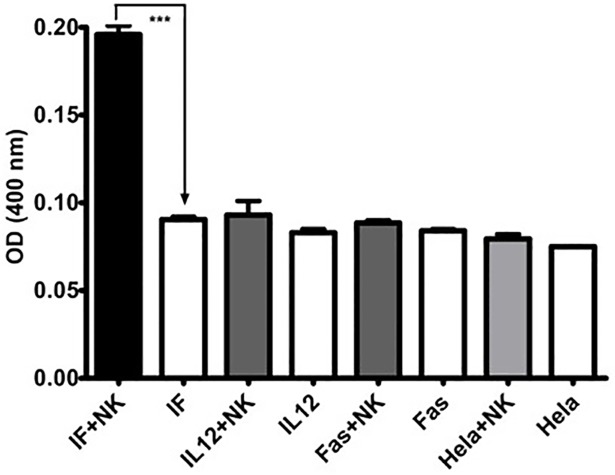
Induction of apoptosis in tumor cells. A total of 2x10^5 cells of Hela cells and lent-clones in triplicates were co-cultured with human NK cells for 24 hours. After the removal of suspension NK92 cells, the caspase 3 activities in the remaining tumor cells were detected using a Caspase 3 Assay Kit (abcam). The statistical analyses were conducted between the corresponding un-induced and induced groups using one-way analysis of variance (ANOVA) with the Tukey’s post test (***p<0.001).

NK92 cells were cocultured with Hela cells transduced with Lent-IF, Lent-IL12 or Lent-Fas for 24 hours to induce apoptosis within the target cells. Caspase 3 assay was performed to analyze the apoptosis in tumor cells. Lent-IF induced significantly higher caspase 3 activity in the transduced Hela cells than all the controls (Fig 6).

## Discussion

The role of the immune system in recognition and elimination of tumor cells has been well recognized. NK92 cells and other killer cells are key players in anti-tumor immune response. NK92 cell activation requires cytokine IL-12. In our previous study, we built a bi-functional fusion gene *IL-12/FasTI* containing mouse *IL-12* sequence and *Fas* transmembrane and intracellular domains and demonstrated its designed activities *in vitro*.

To examine the bi-functional fusion protein’s anti-tumor activity in a therapeutic setting, a lentivirus-based gene delivery system was utilized to deliver the fusion gene construct into human tumor cells. While the raw transfection supernatants contain relatively less viral particles, after concentration, a titer of over 1x10^7^/ml was achieved (Fig 2). After transduction, Hela cells functionally expressed the transduced genes (Fig 3 and Fig 4).

The major purpose of building the bi-functional fusion protein is to achieve local high level expression of IL-12 to activate NK92 cells simultaneously send apoptotic signal to tumor cells. Therefore, human NK92 cells were co-cultured with Hela cells transduced with Lent-IF, Lent-IL12 or Lent-Fas. The activation of NK92 cells after co-culture was determined by the production of IFN-γ (Fig 5A). It is not a surprise that NK92 cells co-cultured with Hela cells transduced with Lent-IF and Lent-IL12 produced a similar amount of IFN-γ because the IL-12 portion of the fusion protein is identical to mouse IL-12. Further studies are needed to find out if Fas signaling is involved in IFN- γ production via NK cells or other cells. Fusion protein IL-12/FasTI clearly demonstrated enhanced overall elimination against tumor cells comparing to IL12 control, while Fas control showed no significant effect on anti-tumor cytotoxicity. (Fig 5B)

Apart from NK92 cell activation, the fusion gene design also involves Fas-induced apoptosis signaling in transmembrane and intracellular domains. The data demonstrated that strong apoptotic signals was sent into tumor cells that express the fusion protein IL12/Fas, not cells that express IL-12 or Fas, when co-cultured with NK92 cells (Fig 6). The data also confirmed a previous study that mouse Fas is able to send apoptotic signals in human cells [14].

In conclusion, lentivirus-based gene expression system was successfully used to deliver fusion gene construct *IL12/FasTI* into tumor cells with potential efficiency, which provided a further step for fusion protein strategies in cancer immune/gene therapies. Furthermore, the fusion gene product *IL-12/FasTI* produced by lentiviral transduction effectively activated killers as indicated by the enhanced production IFN-γ and tumor cell cytotoxicity.

## Supporting information

S1 File Original electrophoresis gel image supplementary to Fig 3. (PPTX)
