# Supplementary material for: Lentiviral delivery of novel fusion protein IL12/FasTI for cancer immune/gene therapy
Source: PLoS One. 2018 Jul 25;13(7):e0201100. doi: 10.1371/journal.pone.0201100 (PMC6059467; doi:10.1371/journal.pone.0201100)
Supplement: S1 File — (PPTX) [file pone.0201100.s001.pptx]

## Slide 1
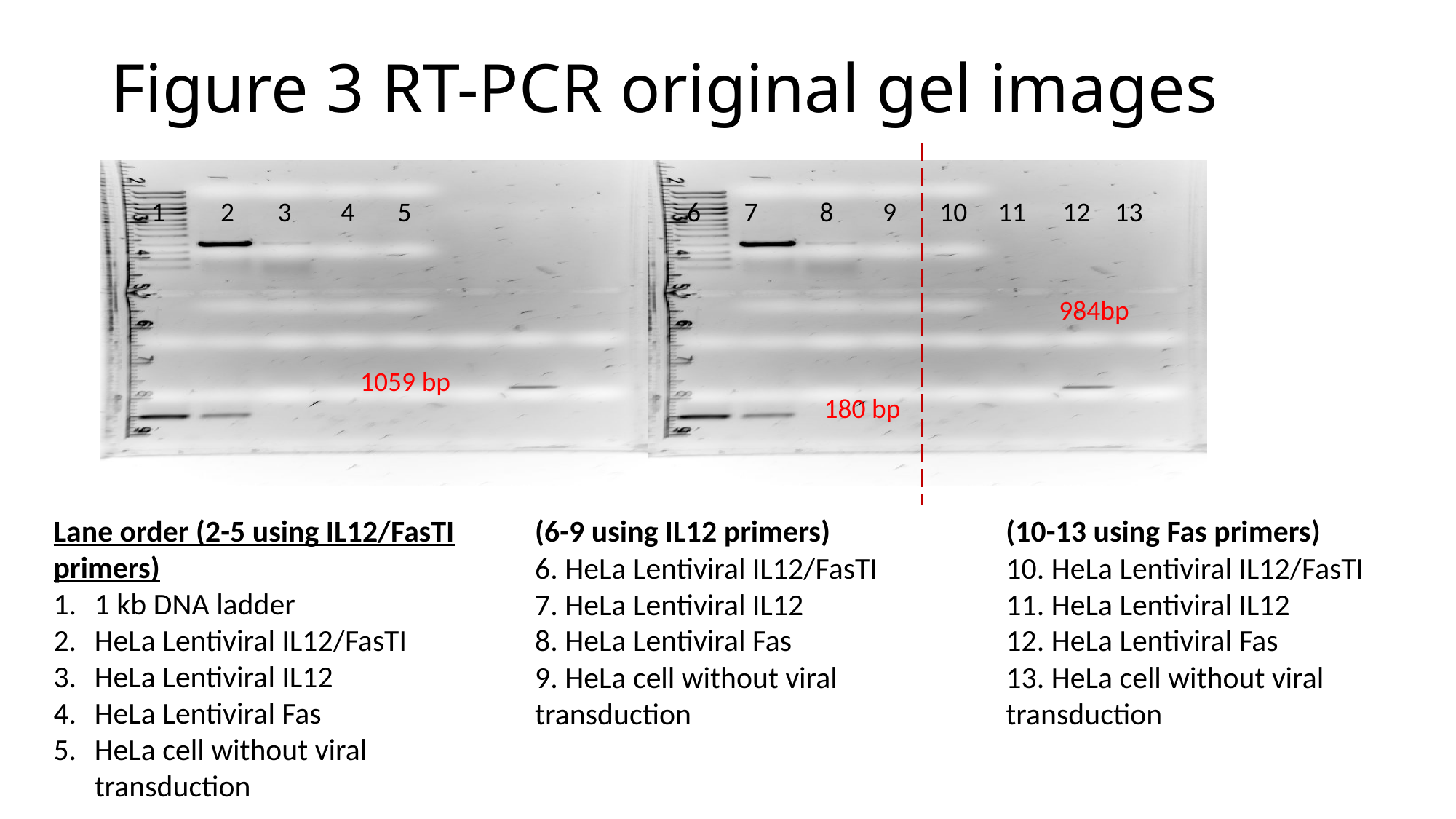

# Figure 3 RT-PCR original gel images
1 2 3 4 5
6 7 8 9 10 11 12 13
984bp
1059 bp
180 bp
1059 bp
Lane order (2-5 using IL12/FasTI primers)
1 kb DNA ladder
HeLa Lentiviral IL12/FasTI
HeLa Lentiviral IL12
HeLa Lentiviral Fas
HeLa cell without viral transduction
(6-9 using IL12 primers)
6. HeLa Lentiviral IL12/FasTI
7. HeLa Lentiviral IL12
8. HeLa Lentiviral Fas
9. HeLa cell without viral transduction
(10-13 using Fas primers)
10. HeLa Lentiviral IL12/FasTI
11. HeLa Lentiviral IL12
12. HeLa Lentiviral Fas
13. HeLa cell without viral transduction

## Slide 2
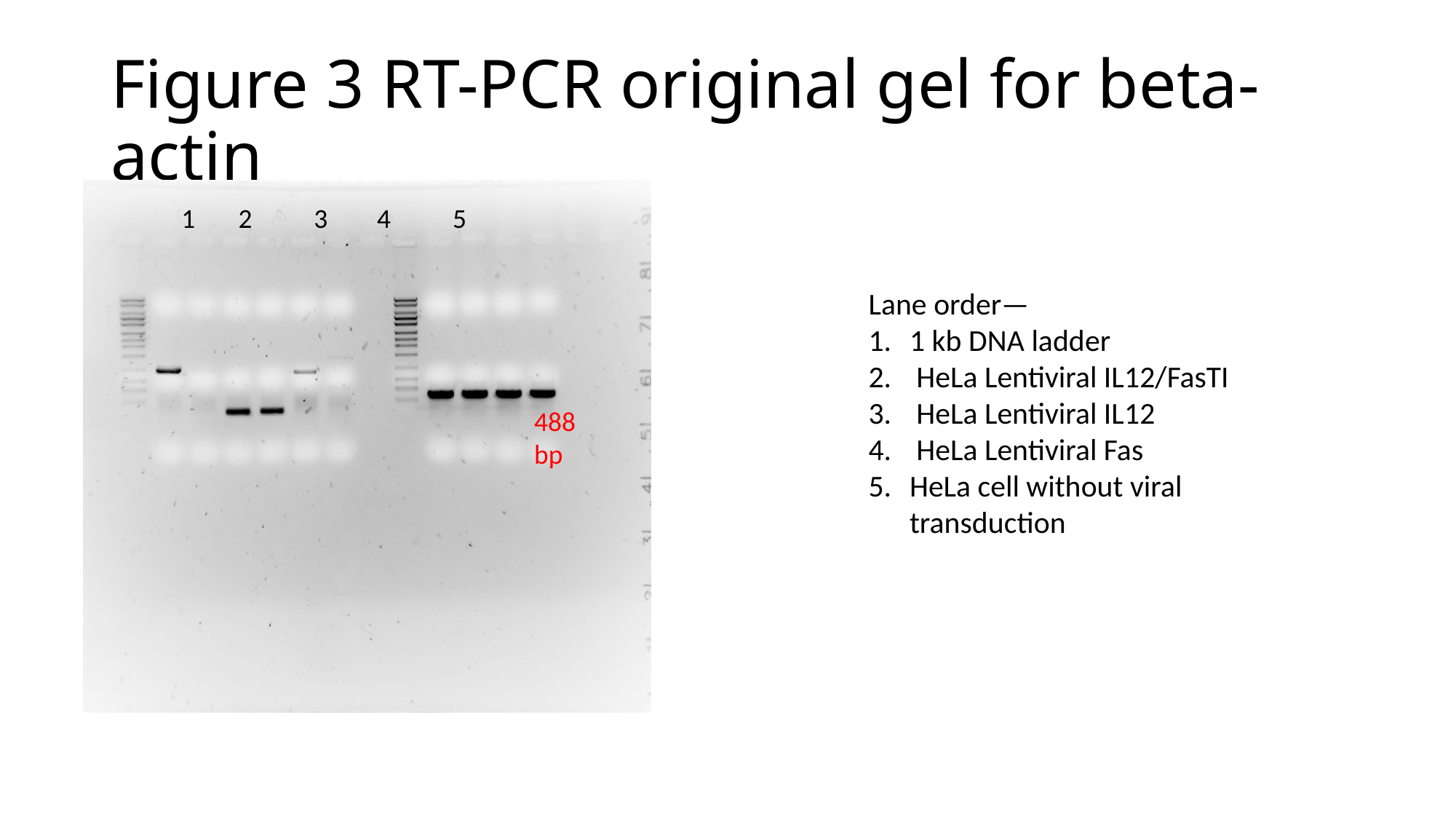

# Figure 3 RT-PCR original gel for beta-actin
 1 2 3 4 5
488 bp
Lane order—
1 kb DNA ladder
 HeLa Lentiviral IL12/FasTI
 HeLa Lentiviral IL12
 HeLa Lentiviral Fas
HeLa cell without viral transduction
